# Factors associated with exclusive breastfeeding in “***Near Miss***” neonates in Brazil

**DOI:** 10.61622/rbgo/2024rbgo59

**Published:** 2024-07-26

**Authors:** Daniele Marano, Theonas Pereira, Yasmin Amaral, Vania De Matos Fonseca, Maria Elisabeth Moreira

**Affiliations:** 1 Instituto Fernandes Figueira Rio de Janeiro RJ Brazil Instituto Fernandes Figueira, Rio de Janeiro, RJ, Brazil.; 2 Centro Universitário UNINOVAFAPI Teresina PI Brazil Centro Universitário UNINOVAFAPI, Teresina, PI, Brazil.; 3 Fundação Oswaldo Cruz Rio de Janeiro RJ Brazil Fundação Oswaldo Cruz, Rio de Janeiro, RJ, Brazil.

**Keywords:** Breastfeeding, Near miss, Infant, newborn

## Abstract

**Objective:**

To assess the association between sociodemographic and perinatal factors and hospital practices to encourage exclusive breastfeeding in *near miss* neonates in maternity hospitals.

**Methods:**

This is a prospective cohort of live births from the survey “To be born in Brazil” conducted between 2011 and 2012. The weighted number of newborns who met the neonatal near miss criteria was 832. Exclusive breastfeeding at hospital discharge and 45 days after delivery were dependent variables of the study. The sociodemographic and perinatal factors of the puerperal women and hospital practices to encourage breastfeeding were independent variables. The data were analyzed with Poisson regression and set with p value<0.05. Is exclusive breastfeeding in neonatal *near misses* associated with factors related to sociodemographic conditions, maternal characteristics and the organization of health services?

**Results:**

Data from 498 women and their children were analyzed. Mothers with incomplete primary education were more likely (36%) to have exclusive breastfeeding (RR: 1.36; 95% CI: 1.06-1.74) at discharge. Women who did not offer the breast to the newborn in the joint accommodation (65%) were less likely to be breastfeeding exclusively (RR: 0.65; 95% CI: 0.56-0.75) at discharge. Variables that increased the probability of exclusive breastfeeding after 45 days of delivery were primiparity (RR: 1.36; 95% CI: 1.08-1.69) and having the newborn in the delivery room (RR: 1.90; 95% CI: 1.12-3.24).

**Conclusion:**

Exclusive breastfeeding in neonatal *near misses* was associated with maternal characteristics and important hospital practices, such as being breastfed in the joint accommodation and the newborn being in the mother’s lap in the delivery room.

## Introduction

Exclusive breastfeeding (EB) allows adequate nourishment of infants in the first six months of life.^(1)^ Over the years, important research has been conducted nationwide to assess the status of breastfeeding in Brazil. Noteworthy are the National Health Demography Survey (PNDS), conducted in 1986, 1996 and 2006, and the National Study of Food and Child Nutrition conducted in 2019, which evaluated 14,584 children under five years of age.^(2,3)^

Based on the results of these studies, it was possible to undertake a temporal analysis of breastfeeding over the last 34 years in the country. The prevalence of EB among children younger than four months increased from 4.7% to 60.0%, representing an absolute increase of 55.3% and a relative increase of 12.8 times.^(3)^A similar pattern was observed in relation to the increase in the prevalence of EB among children below six months of age from 2.9% in 1986 to 45.7% in 2020, an increase of 1.2% per year.^(3)^The increase in the prevalence and duration of breastfeeding observed since the 1970s may have contributed significantly to the improvement of child health indicators in Brazil,^(2)^for instance, reducing hospital admissions due to diarrhea and respiratory infections in children under one year of age countrywide.^(4,5)^

In addition, technological advances in the health domain have strongly impacted the reduction of infant and neonatal mortality in recent decades,^(6)^corroborated by the higher survival rates of newborns (NBs).^(7)^Consequently, the surge of an emerging population group was noticeable, with different new demands of health care putting NBs in a life threatening situation.^(6)^Those NBs present markers of risk of death at birth, but surviving the neonatal period are considered cases of neonatal “*near miss”* (NNM).^(8)^Silva et al,^(8)^ based on data from the survey “To be born in Brazil: National Survey on Delivery and Birth”, defined pragmatic criteria to predict neonatal mortality and assemble the NNM indicator. After testing 19 variables, five constituted the NNM indicator, i.e.: birth weight <1,500 g, Apgar score < 7 in the fifth minute of life, use of mechanical ventilation, gestational age < 32 weeks, and presence of congenital malformations.

The benefits of EB among healthy NBs are well understood and analyzed by numerous studies in Brazil.^(3,5,9)^However, EB is still rarely explored in relation to NNM. Considering the growing population of NNM,^(8)^ the present research aimed to assess the association between sociodemographic and perinatal factors and hospital practices to encourage exclusive breastfeeding in *near miss* neonates in maternity hospitals.

## Methods

The present study consists of a prospective cohort of NB from the research entitled “To be born in Brazil: National Survey on Childbirth”, conducted between 2011 and 2012. The national hospital-based study *Nascer no Brasil*,^(10,11)^conducted between 2011 and 2012, evaluated prenatal, delivery and postpartum care of women who had as pregnancy outcome a newborn alive with any weight or gestational age (GA), or a dead fetus weighing more than 500 grams or GA greater than 22 weeks.

In the first, hospitals with more than 500 deliveries per year were stratified according to the five macro-regions of the country, location (capital or countryside) and type of service (public, mixed or private). Then 266 hospitals with probability of selection proportional to the number of deliveries in each of the strata were selected. In the second stage, the number of days needed to interview 90 puerperal women in each hospital was defined using an inverse sampling method. In the third, eligible women were selected on each day of fieldwork. The number of postnatal women sampled was 23,940, distributed in 191 municipalities

Data collection included face-to-face interviews conducted during hospitalization; extraction of data from the prenatal card, when available; extraction of data from maternal and newborn medical records after hospital discharge; and two telephone interviews after hospital discharge. More information about the sampling process and design of the study *Nascer no Brasil* can be found at Leal et al.^(10)^and Vasconcellos et al.^(11)^In this analysis, we used data obtained in the hospital interview and in the prenatal card.

In the present study, all NBs who survived the neonatal period and who presented at least one of the death risk predictors of the NNM index were considered a case. For NNM, we used the classification of Silva et al.,^(8)^and the set of neonatal near miss indicators consisting of five variables (birth weight of less than 1,500 g, an Apgar score of less than 7 in the 5th minute of life, use of mechanical ventilation, gestational age of less than 32 weeks, and congenital malformations) was able to identify situations with a high risk of neonatal death.

The definition of the dependent variable EB was established according to the World Health Organization (WHO), that is, when the child receives only breast milk, direct from the breast or extracted, or human milk from another source, without other liquids or solids, except for drops or syrups containing vitamins, oral rehydration salts, mineral supplements or medications.^(12)^Regarding the construction of the second dependent variable, EB 45 to 90 days after delivery, the puerperal should have been offered only breast milk in the last 24 hours and no other food or liquid as defined above.^(12)^

The independent variables of this study were the sociodemographic factors of the puerperal women (maternal age: 12 to 19 years, 20 to 34 years, 35 years and older; maternal education in completed years: incomplete elementary school, complete elementary school, incomplete higher education, complete higher education; economic class: A+B, C, D+E; marital status: without a partner, with a partner); perinatal factors (type of delivery: vaginal, cesarean section; primiparous: no, yes; adequacy of prenatal care: no, yes; hospital/maternity location: in the capital, outside the capital; maternal labor: no, yes; premature delivery: no, yes; classification of prematurity: not premature, very premature and extreme; type of hospital unit: public, private; place of hospitalization of the NB: joint accommodation, neonatal ICU. In addition, we also evaluated the hospital practices to encourage breastfeeding (in the delivery room: the NB was placed to breastfeed, the NB was placed in the mother’s lap, the mother only saw the NB, the mother had no contact with the NB; in the joint accommodation: the NB went to joint accommodation, the mother offered the breast to the NB in the joint accommodation, the mother offered the breast to the NB in the delivery room, the NB received another liquid other than breast milk).^(11)^

To assess the adequacy of prenatal care, the gestational trimester at the beginning of prenatal care (PN), the total number of consultations corrected by gestational age at the time of delivery, routine examinations performed and the advice provided on the reference maternity for childbirth care were measured. Prenatal care was considered appropriate when it was initiated up to the 12th gestational week,^(12-14)^as recommended by the Stork Network. or more details on the analysis of the adequacy of prenatal care, refer to Domingues et al.^(13)^The classification of economic status estimates the purchasing power of people and families regarding the possession of assets and education level of the head of household.^(15)^This classification comprises five categories, from A to E, as well as their subgroups (A1, A2, B1, B2, C1, C2, D1, D2, E). Given the small number of women in classes A and E, the economic classes were regrouped into three categories (A + B; C; D + E).^(15)^

Prematurity was evaluated using the classification proposed by the World Health Organization (WHO): extremely preterm infants (<28 weeks), very premature infants (28-31 weeks) and moderate infants (32-36 weeks of gestation).^(16)^The other independent variables analyzed in this study are self-explanatory. For data analysis, we estimated the absolute and relative frequencies of the risk predictor variables. Bivariate analysis was carried out to verify potential associations between EB at hospital discharge and 45 to 90 days after delivery (dependent variable) and independent variables (sociodemographic and perinatal factors and hospital practices to encourage breastfeeding). The variables with a p value <0.20 in the bivariate analysis were selected individually to be included in the multivariate analysis. The strength of association between the dependent and independent variables was tested with Poisson regression analysis, and the results were expressed as risk ratios (RR), with their associated confidence intervals set at 95%. Only the variables with a *P* value <0.05 in the multivariate analysis were maintained in the final model.

The larger study was approved by the Research Ethics Committee (CEP) of the Sérgio Arouca National School of Public Health, Oswaldo Cruz Foundation (ENSP/Fiocruz), under opinion no. 92/10. For the purpose of this study, analysis and approval by the CEP of the National Institute of Women, Children and Adolescents Fernandes Figueira, Fundação Oswaldo Cruz (IFF/Fiocruz) was waived. This study protocol has been registered in the ethics committee of the Instituto Fernandes Figueira - IFF/FIOCRUZ, with opinion number 3.376.235, CAAE: 14248719.1.0000.5269.

## Results

Among the 23,837 NB, only 832 met the criteria to be classified as NMN cases. Of these, 518 had recorded information about EB, 70.5% were discharged with EB, and only 40% remained on EB 45 to 90 days after delivery. For the NNM, having gestational age <32 weeks and congenital malformations reduced the probability of EB at hospital discharge. Low birth weight (< 1,500 g) reduced the probability of EB at 45 to 90 days after delivery (Table 1). EB at hospital discharge was more frequent among mothers aged 12 to 19 years (81.5%), with incomplete primary education (77.2%), multiparous women (74.3%) and those who did not have adequate prenatal care (71.7%). This was also found among nonpremature NNM (83%) and those who proceeded to the joint accommodation (88.4%). On the other hand, EB 45 to 90 days after delivery was more frequent among mothers over 35 years of age (43%), those who completed high school (43.5%), multiparous women (46.4%) and those who underwent adequate prenatal care (41.4%) (Table 2).

**Table 1:** Exclusive breastfeeding at hospital discharge and 45 to 90 days after delivery among neonatal “Near Miss”

Variables	Exclusive breastfeeding at discharge			Exclusive breastfeeding 45-90 days after delivery		
	No (%)	Yes (%)	p-value	No (%)	Yes (%)	p-value
Birthweight<1500 g (n=495)						
No	72.1	81.7	0.130	73.9	86.5	0.006
Yes	27.9	18.3		26.1	13.5	
Gestational age <32 weeks (n=498)						
No	58.0	76.7	0.010	82.2	84.6	0.744
Yes	42.0	23.3		16.8	15.4	
Congenital malformation (n=498)						
No	70.1	57.1	0.019	62.9	58.0	0.399
Yes	29.9	42.9		37.1	42.0	
Mechanical ventilation (n=337)						
No	49.6	56.5	0.512	52.6	56.2	0.621
Yes	50.4	43.5		47.4	43.8	
Apgar>7 in the 5th minute (n=485)						
No	87.9	84.6	0.221	83.2	84.6	0.744
Yes	12.1	15.4		16.8	15.4	

**Table 2:** Sociodemographic and perinatal variables and exclusive breastfeeding at discharge and 45-90 days after delivery among neonatal “Near Miss”

Variables	Total	Exclusive breastfeeding at discharge			Exclusive breastfeeding 45-90 days after delivery		
		Yes	RR (95% CI)	Valor p	Sim	Yes	RR (95% CI)
Mother’s age (years) (n=498)						(n=498)	
12 to 19	21.8	81.5	1.20 (1.02-1.41)	0.025	29.7	0.70 (0.50-0.96)	0.027
20 to 34	63.9	67.9	1.0	-	42.7	1.0	-
≥ 35	14.2	65.5	0.96 (0.72-1.29)	0.807	43.0	1.01 (0.66-1.53)	0.970
Mother’s schooling (n=497)						(n=497)	
ES* incomplete	23.2	77.2	1.37 (1.05-1.79)	0.022	37.9	1.30 (0.73-2.29)	0.371
ES* complete	26.8	73.5	1.30 (0.98-1.72)	0.064	39.6	1.35 (0.79-2.33)	0.271
HS**complete	42.0	67.4	1.20 (0.87-1.64)	0.267	43.5	1.49 (0.81-2.74)	0.200
HS** complete and more	8.0	56.4	1.0	-	29.2	1.0	-
Economic class (n=493)						(n=493)	
Class A + B	23.3	66.9	1.0	-	35.8	1.0	-
Class C	57.7	68.5	1.02 (0.86-1.22)	0.792	42.6	1.19 (0.83-1.70)	0.346
Class D + E	19.0	80.0	1.20 (1.02-1.41)	0.030	38.6	1.08 (0.71-1.64)	0.733
Marital status (n=497)						(n=497)	
No partner	20.6	74.9	1.08 (0.88-1.32)	0.446	33.2	0.80 (0.55-1.16)	0.230
With partner	79.4	69.3	1.0	-	41.8	1.0	-
Type of delivery (n=485)						(n=485)	
Vaginal	39.3	76.4	1.0	-	43.0	1.0	-
Caesarean	60.7	67.2	0.88 (0.70-1.10)	0.255	38.5	0.90 (0.69-1.17)	0.418
Primiparous (n=498)						(n=498)	
No	47.9	74.3	1.0	-	46.4	1.0	-
Yes	52.1	67.0	0.90 (0.75-1.08)	0.266	34.0	0.73 (0.56-0.96)	0.023
Adequate prenatal care (n=498)						(n=498)	
No	45.0	71.7	1.03 (0.83-1.28)	0.772	38.1	0.92 (0.67-1.26)	0.608
Yes	55.0	69.5	1.0	-	41.4	1.0	-
Place of delivery (n=498)						(n=498)	
Capital	55.9	74.3	1.0	-	45.1	1.0	-
Not in a capital	44.1	65.7	0.88 (0.75-1.04)	0.130	33.4	0.74 (0.55-1.00)	0.051
Maternal work (n=497)						(n=497)	
No	56.4	75.1	1.0	-	36.8	1.0	-
Yes	43.5	64.3	0.86 (0.70-1.04)	0.128	44.2	1.20 (0.95-1.52)	0.130
Premature delivery							
No	46.4	81.9	1.0		45.6	1.0	
Yes	53.6	59.8	0.72 (0.62-0.85)	<0.001	34.6	0.76 (0.50-1.15)	0.192
Prematurity classification							
No	42.1	83.0	1.0		45.7	1.0	
Premature	29.2	65.8	0.79 (0.69-0.91)	0.001	41.8	0.91 (0.71-1.17)	0.482
Very premature	26.9	57.8	0.70 (0.54-0.89)	0.004	31.5	0.69 (0.31-1.55)	0.369
Extreme premature	1.9	46.3	0.56 (0.19-1.65)	0.291	-	-	-
Type of hospital unit							
Public	48.7	72.4	1.0		43.2	1.0	
Private	51.3	68.8	0.95 (0.80-1.12)	0.541	36.8	0.85 (0.62-1.17)	0.325
Location of NB* admission							
Joint accommodation	29.0	88.4	1.0		43.2	1.0	
Neonatal ICU	71.0	63.2	0.72 (0.62-0.82)	<0.001	38.6	0.89 (0.64-1.25)	0.504

*ES - elementary school; **HS - high school; ***NB - newborn

In the bivariate analysis, the variables associated with EB at hospital discharge were being an adolescent mother (12 to 19 years) (RR: 1.20; 95% CI: 1.02-1.41), incomplete elementary school (RR: 1.37; 95% CI: 1.05-1.79), belonging to social class D+E (RR: 1.20; 95% CI: 1.02-1.41), premature birth (RR: 0.72; 95% CI: 0.62-0.85) and staying in the neonatal ICU (RR: 0.72; 95% CI: 0.62-0.82). The variables associated with EB 45 to 90 days after delivery were being an adolescent mother (RR: 0.70; 95% CI: 0.50-0.96), primiparity (RR: 0.73; 95% CI: 0.56-0.96) and not living in the capital (RR: 0.74; 95% CI: 0.55-1.00) (Table 2). Among the mothers who were in the joint accommodation, 89.7% left practicing EB at discharge, and 90.6% breastfed in the delivery room. The women who offered the breast to the NB in the joint accommodation (46.3%) maintained EB 45 to 90 days after delivery (Table 3). In the bivariate analysis, the variables related to hospital practices associated with EB at hospital discharge were NBs not going to the joint accommodation with the mother (RR: 0.72; 95% CI: 0.64-0.82); mother did not offer the breast to the NB in the joint accommodation (RR: 0.64; 95% CI: 0.56-0.74), and the NB received liquid other than mother’s milk (RR: 0.78; 95% CI: 0.63-0.96). No variable was associated with EB 45 to 90 days after delivery (Table 3). From the bivariate analysis for the EB outcome at hospital discharge, schooling, location of delivery outside the capital, mother did not offer the breast to the NB n the joint accommodation and location of hospitalization were included in the multivariate regression model. For EB 45 to 90 days after delivery, we included the variables primiparity, location of delivery outside the capital and with the NB in the delivery room (Table 4). The multivariate analysis showed that mothers with incomplete elementary school (RR: 1.36; 95% CI: 1.06-1.74) were 36% more likely to maintain EB at discharge. The mother not offering the breast to the NB in the joint housing reduced the probability of EB by 65% when discharged (RR: 0.65; 95% CI: 0.56-075). The variables associated with EB 45 to 90 days after delivery were primiparity (RR: 1.36; 95% CI: 1.08-1.69) and the NB staying in the mother’s lap in the delivery room, a procedure that almost doubled (90%) the probability of this outcome (RR: 1.90; 95% CI: 1.12-3.24) (Table 4).

**Table 3:** Hospital practices. exclusive breastfeeding at discharge and at 45-90 days after delivery among neonatal near misses (NNMs)

Variables	Total	Exclusive breastfeeding at discharge			Exclusive breastfeeding 45-90 days after delivery		
		Yes	RR (95% CI)	p-value	Yes	RR (95% CI)	p-value
In the delivery room (n=497)						(n=497)	
Put to suckle	1.8	83.4	1.0	-	34.9	1.0	-
Put the NB*on her lap	15.9	70.4	0.84 (0.52–1.38)	0.497	66.3	1.89 (0.72-4.98)	0.192
Just saw the NB*	63.4	72.2	0.86 (0.65 – 1.15)	0.314	35.1	1.01 (0.41-2.47)	0.991
Had no contact with the NB*	18.9	63.6	0.76 (0.52 – 1.11)	0.161	35.1	1.00 (0.36-2.76)	0.993
NB* went to joint accommodation (n=497)						(n=497)	
No	77.8	65.7	0.72 (0.64 - 0.82)	<0.001	37.9	0.80 (0.59-1.08)	0.142
Yes	22.2	89.7	1	-	47.4	1	-
Mother offered breast to NB* in joint accommodation (n=498)						(n=498)	
No	60.8	57.9	0.64 (0.56 – 0.74)	<0.001	36.3	0.78 (0.55-1.11)	0.167
Yes	39.2	89.6	1.0	-	46.31	-	
Mother offered breast to NB* in delivery room (n=498)						(n=498)	
No	85.8	89.7	0.99 (0.87 – 1.12)	0.875	45.9	0.92 (0.57-1.49)	0.733
Yes	14.2	90.6	1.0	-	49.9	1.0	-
NB* received milk or liquid other than breast milk (n=498)						(n=498)	
No	45.4	83.0	1.0	0.009	45.8	1.0	-
Yes	54.6	64.7	0.78 (0.63 – 0.96)		42.7	0.93 (0.71-1.23)	0.611

*NB - Newborn

**Table 4:** Multivariate model among sociodemographic, perinatal variables, hospital practices, exclusive breastfeeding at hospital discharge and 45-90 days after delivery among neonatal near misses (NNM)

	Exclusive breastfeeding at discharge			Exclusive breastfeeding 45-90 days after delivery		
	RR	95% CI	p-value	RR	95% CI	p-value
Schooling						
ES* incomplete	1.36	1.06 –1.74	0.017	-	-	-
ES* complete	1.26	0.97 –1.64	0.087	-	-	-
HS**complete	1.22	0.91 –1.64	0.177	-	-	-
HS** complete and more	1.0	-	-	-	-	-
Mother offered breast to NB*** in joint accommodation						
No	0.65	0.56-0.75	<0.001	-	-	-
Yes	1	-	-	-	-	-
Location at NB*** admission						
Joint accommodation	1.0	-	-	-	-	-
Neonatal ICU	0.94	0.81 – 1.08	0.381	-	-	-
Primiparous						
No	-	-	-	1.0	-	-
Yes	-	-	-	1.36	1.08 – 1.69	0.008
In the delivery room						
NB breastfed	-	-	-	1.0	-	-
NB was in her lap	-			1.90	1.12- 3.24	0.017
Mother only saw the NB	-	-	-	1.01	0.63 - 1.62	0.954

*ES - elementary school; * HS - high school; ***NB - newborn

## Discussion

The present study found that of the 70.5% NBs discharged with EB, only 40% continued to be exclusively breastfed 45 to 90 days after delivery. Although the percentages of EB at hospital discharge found in the present study fall below the WHO recommendation (90% at hospital discharge), they remain within the parameters considered acceptable.^(16)^ It is noteworthy that this recommendation considers all deliveries and does not exclude clinical situations of newborns. This points to the need for new recommendations to assess the adequacy of EB for newborns with unfavorable life conditions, such as NNM.

Given the scarcity of publications on the factors associated with NNM among EB babies, the comparisons of our findings with the literature were based on the variables that make up the NNM indicator. Gestational age < 32 weeks and congenital malformations were associated with lower frequencies of EB (23.3% and 42.9%, respectively) at hospital discharge. We also found that the lower the gestational age was, the lower the probability of EB at hospital discharge. Regarding prematurity, in a cohort from southern Brazil with 113 NB, 81.4% maintained EB at discharge, and 66.4% maintained EB between seven and 15 days subsequently.^(17)^This difference in the percentage of EB between the present study and the cohort can be partially explained using different cutoff points for classification of prematurity, 32 weeks versus 37 weeks, as currently defined by the WHO.^16^It is also to be emphasized that the cohort comes from a maternity services set up that since its creation has established practices of promotion, protection, and support for breastfeeding. Furthermore, the maternity hospital had a human milk collection room and a partner milk bank and implemented the three stages of the kangaroo method.^(17)^

The sampling of hospitals to participate in the research “To be born in Brazil” did not consider as an inclusion criterion the accreditation as a Child-Friendly Hospital. Among the sociodemographic factors associated with NNM among EB, we observed that women who did not complete primary education had a 36% higher probability of adherence to EB at discharge than those with complete high school education. Some studies have pointed out that mothers with low schooling tended to breastfeed exclusively for a longer timeand in the first hour of life of the NB compared to those with postsecondary education.^(18-21)^ One possible explanation could be the greater availability of these mothers with low schooling, given that 80% did not work outside the home and they usually belong to low-income families,^(3)^ and EB does not require increased spending for the family. This condition may also have contributed positively to the promotion of EB, because according to Baptista et al.,^(22)^the increasing participation of women in the labor market has elevated the incidence of early weaning.

Another issue deserving attention as a factor contributing to the increase in EB duration, is the higher attendance of women with low schooling (<8 years) at health facilities, favoring the opportunities for guidance on EB. Among the perinatal factors, being primiparous increased the probability of EB 45 to 90 days after delivery. Some studies have already shown that this group of women is more likely to start breastfeeding early, but the timespan of EB tends to be shorter than that of multiparous women. Most primiparous women in the present study, 76.6%, lived with their partners (data not shown in table). A survey of 146 mothers from 2009 to 2013 in Indiana, USA, showed that 97% of mothers who received support from their partners immediately after delivery maintained breastfeeding after hospital discharge, and 26% maintained EB until six months. Among those who did not have such support, the prevalence was much lower (10.1%).^(23-26)^

Social and family support have been cited as determinants for EB, and fathers and grandmothers are the most influential in this relationship. Therefore, breastfeeding should be a social practice and requires expanded actions of promotion, protection, and support beyond women’s sphere, in which health professionals,^(27)^the family and the overall community ought to be included.^(25)^

Joint accommodation is a hospital system allowing the NB to remain with the mother in the same room, 24 hours a day, immediately after birth, if possible, until hospital discharge, step seven of the “Ten Steps to Successful Breastfeeding”. Therefore, it is considered a necessary practice for the implementation of the Child-Friendly Hospital Initiative.^(28)^ Of all newborns who stayed in the joint accommodation, 88.4% sustained EB at discharge, while the prevalence among those who went to the intensive care unit (ICU) was 63%. Therefore, being admitted to the ICU reduced the probability of EB at hospital discharge by 28%. Thus, 77.8% of the NB were not assigned to the joint accommodation with the mother, and of those who were, 71.1% did not receive another milk or liquid in the hospital. For the newly born who went to ICU/intermediate care, 68.5% received milk or liquid other than mother’s milk in the hospital (data not included in the table).

In a cross-sectional study conducted in a Brazilian capital and comprising 1,170 mother and child pairs, the prevalence of EB in the first hour of life was higher among those who remained in joint accommodation (83.8%).^(28)^ A systematic Cochrane review revealed that the prevalence of EB, up to the fourth day before hospital discharge, was higher among women who remained in joint accommodation than among those who did not.^(24)^

Although the present study did not evaluate the reasons for not offering the breast to NNM in the joint accommodation, the lack of this procedure may have had repercussions on the establishment of breastfeeding, leading to a possible explanation for this outcome. It is noteworthy that NNM should receive a differentiated attention regarding the routines and techniques of the multidisciplinary health team, ensuring in particular skin-to-skin contact, stimulus of periodic milking with subsequent feeding with the mother’s milk and, whenever possible, breastfeeding on mother’s lap. A systematic review pointed out that the practice of skin-to-skin contact can increase the length of breastfeeding in the first four months and contribute to EB.^(27)^

We also observed that placing the NB on the mother’s lap in the delivery room increased the probability of sustaining EB, both at discharge and 45 to 90 days after delivery. In the present study, this may be due to the characteristics of the NNM sample; only 15.9% of newborns had skin-to-skin contact with their mothers in the delivery room. However, a study conducted by Maastrup and Greisen,^(29)^which evaluated extremely premature newborns with a mean gestational age at birth of 25 weeks and 4 days, showed that 100% of the clinically stable newborns maintained adequate skin temperature and physical stability when establishing skin-to-skin contact. The skin-to-skin contact of the NB with the mother creates favorable conditions for maintaining breastfeeding.^(24)^

Exclusive breastfeeding in neonatal *near misses* was associated with maternal characteristics and important hospital practices, such as being breastfed in the joint accommodation and the newborn being in the mother’s lap in the delivery room. So these results reinforce the need to promote practices that create an ideal environment for breastfeeding, from the beginning of labor up to discharge from the hospital, even if newborns have life-threatening situations.^(26)^ Special attention should be given to the preparation of discharge and to the requirements of a maternal support network.^(26)^ Therefore, keeping the mother and the NB together is a prerequisite for early onset of breastfeeding, as their separation, even for a very brief period, results in breastfeeding delays.^(30)^

## Conclusion

Exclusive breastfeeding in neonatal *near misses* was associated with maternal characteristics and important hospital practices, such as being breastfed in the joint accommodation and the newborn being in the mother’s lap in the delivery room.
